# Screening for Combination Cancer Therapies With Dynamic Fuzzy Modeling and Multi-Objective Optimization

**DOI:** 10.3389/fgene.2021.617935

**Published:** 2021-03-31

**Authors:** Simone Spolaor, Martijn Scheve, Murat Firat, Paolo Cazzaniga, Daniela Besozzi, Marco S. Nobile

**Affiliations:** ^1^Department of Informatics, Systems and Communication, University of Milano-Bicocca, Milan, Italy; ^2^Department of Industrial Engineering & Innovation Sciences, Eindhoven University of Technology, Eindhoven, Netherlands; ^3^Department of Human and Social Sciences, University of Bergamo, Bergamo, Italy; ^4^SYSBIO/ISBE.IT Research Centre of Systems Biology, Milan, Italy; ^5^Bicocca Bioinformatics Biostatistics and Bioimaging Centre (B4), Milan, Italy

**Keywords:** fuzzy modeling, multi-objective optimization, global optimization, cancer, therapeutic targets, combination chemotherapy

## Abstract

Combination therapies proved to be a valuable strategy in the fight against cancer, thanks to their increased efficacy in inducing tumor cell death and in reducing tumor growth, metastatic potential, and the risk of developing drug resistance. The identification of effective combinations of drug targets generally relies on costly and time consuming processes based on *in vitro* experiments. Here, we present a novel computational approach that, by integrating dynamic fuzzy modeling with multi-objective optimization, allows to efficiently identify novel combination cancer therapies, with a relevant saving in working time and costs. We tested this approach on a model of oncogenic K-ras cancer cells characterized by a marked Warburg effect. The computational approach was validated by its capability in finding out therapies already known in the literature for this type of cancer cell. More importantly, our results show that this method can suggest potential therapies consisting in a small number of molecular targets. In the model of oncogenic K-ras cancer cells, for instance, we identified combination of up to three targets, which affect different cellular pathways that are crucial for cancer proliferation and survival.

## 1. Introduction

Combination therapy has become a fundamental tool in the fight against several types of cancers. The treatment of patients with combined therapeutic agents gives several advantages with respect to single drug treatments. Targeting different cellular pathways in a synergistic or additive manner increases the efficacy and the outcome of the therapy, by inducing programmed cell death in tumor cells, and by reducing tumor growth, metastatic potential or the risk of developing drug resistance (Mokhtari et al., 2017; Palmer and Sorger, 2017). However, the identification of effective combination therapies is complex, costly and time consuming, with the major bottleneck being represented by the *in vitro* experimental phase necessary to identify potential drug targets (Ilag et al., 2002). This is further hindered by the lack of a complete understanding of the cellular mechanisms involved in the onset and development of cancer, which are characterized by emergent, non-linear dynamic behaviors that arise from the interactions between a huge number of different molecules and processes within the cell (Gottesman et al., 2016).

Mathematical models of combination cancer therapies have the makings of predicting potentially better treatment outcomes based on novel combinations of drug regimens (see e.g., Wang and Deisboeck, 2014; Bulusu et al., 2016; Malinzi et al., 2021). Specifically, in the context of targeted therapies, mathematical and computational approaches are becoming more and more relevant to face various challenges related to drug target identification (Smith, 2003; Brubaker and Lauffenburger, 2020) and, in particular, in the context of precision oncology, where different types of data are integrated and analyzed to identify effective treatments for cancer (Senft et al., 2017). Among the computational methods currently available in the literature (Barillot et al., 2012), dynamic models proved to be notably successful in drug target identification (Smith, 2003; Brubaker and Lauffenburger, 2020). Their integration with experimental data can elucidate new emergent properties in physiological and pathological conditions, reveal possible counter-intuitive mechanisms, and predict the response to an extensive number of perturbations, therefore reducing the duration and costs of the experimental research (Kitano, 2002; Faeder and Morel, 2016). Several types of dynamic models were employed to study and explore drug targets in cancer cells (Sun and Hu, 2018): the most common include ordinary or partial differential equations (Jackson and Byrne, 2000; Kirouac et al., 2017), and stochastic and Markovian processes (Komarova, 2006; Haeno et al., 2012). Agent-based models (Vaidya et al., 2019) and logic-based models (Flobak et al., 2015; Morris et al., 2016) are also present in the literature. Despite the plethora of available modeling formalisms, the investigation of complex cellular systems, such as cancer cells, still presents some shortcomings. The definition of computational models is often limited by the lack of quantitative information (e.g., kinetic parameters or copy number of proteins), and the analysis of all system's perturbations might be computationally burdensome, due to the size of the search space or the presence of a plethora of locally optimal solutions.

To overcome these drawbacks, in this paper we propose a novel computational strategy that combines dynamic fuzzy modeling (Nobile et al., 2020) and evolutionary multi-objective optimization (Deb, 2007) to identify new therapeutic targets in cancer cells. Dynamic fuzzy models (DFMs) make use of fuzzy logic (Yen and Langari, 1999) to represent complex systems composed by heterogeneous components, taking into account qualitative and semi-quantitative data, and providing an interpretable description of the system under investigation. The simulation of DFMs allows to predict the evolution over time of the state of the system, without the need of precise and quantitative kinetic information. Evolutionary multi-objective optimization is here coupled with DFMs to identify combinations of the system's components that need to be perturbed in order to lead to an expected behavior; in this work, we exploit the Non-dominated Sorting Genetic Algorithm (NSGA-II) (Deb et al., 2002). As a case study, we considered the DFM presented in Nobile et al. (2020), which describes oncogenic K-ras cancer cells characterized by a marked “Warburg effect,” and grown in glucose depletion. This DFM was previously validated against data obtained from both mouse fibroblasts transformed by oncogenic K-ras expression (NIH3T3 K-ras cells) and a human K-ras-mutated breast cancer cell line (MDA-MB-231) (Nobile et al., 2020). In this work, we use NSGA-II to identify the combination of components of this DFM that represent potential therapeutic targets, and that satisfy the following goal: maximize the induction of apoptosis and minimize the initiation of necrotic processes, while minimizing the number of perturbations.

The integration between DFM and NSGA-II allowed to automatically and efficiently explore a search space consisting in more than 10^8^ possible perturbations. Our results show that this approach was able to find potential novel therapeutic treatments consisting of combinations of no more than 3 cellular targets, in addition to a set of perturbations that were already validated in the literature. We envision that such a computational approach could be used to guide the development of novel combination therapies in cancer cells, but also in different multi-factorial diseases (e.g., neurodegeneration).

## 2. Methods

In this section, we describe the methods used to predict novel combinations of therapeutic targets: dynamic fuzzy modeling (section 2.1), and multi-objective optimization by means of NSGA-II (section 2.2).

### 2.1. Dynamic Fuzzy Modeling

Cells are complex systems whose functioning depends on the interplay among a huge number of molecules involved in different processes (e.g., gene regulation, signal transduction, metabolic pathways). In principle, detailed mechanistic models of these interactions could be exploited to simulate the emergent cellular dynamics (Spolaor et al., 2019) by means of, e.g., differential equations, Markov chains, or integrated multi-paradigm models as proposed by Karr et al. (2012). However, mechanistic modeling is generally unfeasible because of the lack of precise quantitative data related to kinetic parameters and molecular amounts, which are generally difficult or even impossible to measure *in vivo*. As a consequence, it is hard to find an accurate model parameterization, which represents an indispensable piece of information to faithfully reproduce the system's behavior. In addition, biological knowledge is often described in qualitative terms, using natural language expressions such as “when the concentration of glucose is high, glycolysis is highly active.” Complex biological systems are also characterized by multiple scales of temporal, spatial and functional organization, leading to extremely high computational efforts for their simulation and analysis (see e.g., Karr et al., 2012; Tangherloni et al., 2017).

Dynamic Fuzzy Models (DFMs) have been introduced to tackle all these issues and to provide a qualitative description of all the possible states that any system's component can assume over time (Yen and Langari, 1999; Aldridge et al., 2009; Nobile et al., 2020). Thanks to DFMs, it is possible to account for the heterogeneous nature of biological systems, and to bypass the problem of assigning quantitative data to the state of activity and functioning of whole cellular processes. As such, DFMs can be efficiently used to describe and simulate the emergent behavior of systems characterized by uncertainty. Moreover, DFMs exploits knowledge-based rules to harmonize data obtained from multiple sources (omics data, microscopy imaging, etc.), and integrate different data types (protein and processes activation/inhibition, post-translational modifications, etc.).

In DFMs, a directed graph is employed to represent the set of the system's components and their functional interactions (e.g., positive or negative regulations); a linguistic variable and a list of linguistic terms (e.g., low, medium, and high) are used to describe each system's component; membership functions are associated with each linguistic term to handle the intrinsic uncertainty of the state of the variables. The interactions among the variables of a DFM are modeled using fuzzy rules, that is, conditional statements expressed in natural language, written in the form:


IF x IS a THEN y IS b.


The left-hand side of a fuzzy rule is called antecedent: it is a predicate involving linguistic variables (in the example, the system's component x) and their associated linguistic terms (in the example, the linguistic term a). The right-hand side of a fuzzy rule is called consequent: it represents the conclusion drawn given the premises in the antecedent. In the example, y represents an output variable, while b could be a fuzzy set (e.g., in the case of Mamdani fuzzy inference systems Mamdani and Assilian, 1975), a real number (e.g., in the case of 0-order Sugeno fuzzy inference systems), or a function of the variables appearing in the antecedent (e.g., in the case of higher-order Sugeno fuzzy inference systems) (Sugeno, 1985).

The set of linguistic variables and fuzzy rules constitute together a Fuzzy Inference System (FIS), and the process of calculating the rules' output is known as fuzzy inference (Yen and Langari, 1999; Yager and Zadeh, 2012). A DFM can be considered as a fuzzy network (FN) (Gegov, 2010), that is, a network of interacting FISs. In a FN, nodes represent linguistic variables, while the connections among them represent fuzzy rules, whose outputs are fed as variable inputs to downstream linguistic variables. Thus, a DFM allows to represent the system's components and their mutual regulations, including feedback loops, and provides a qualitative description of the underlying mechanisms driving the overall behavior of the system. Given the fact that FISs were designed to deal with uncertainty in their input values, the variables of a DFM are inherently robust against small perturbations of their input values (Johansen, 1994). Nevertheless, even if each FIS is locally robust to small perturbations of their input values, the robustness of the global behavior of a DFM with respect to the addition and/or deletion of interactions is difficult to predict, since it depends on the actual topology of the system under investigation.

The linguistic variables belonging to a DFM can be partitioned into two disjoint sets of outer and inner variables:

- The outer variables can be classified as *input* or *output* variables: input variables correspond to the components that trigger the dynamics of the system and appear only in the antecedents of rules, while output variables represent the components of interest for the analysis of the system (for instance, some experimentally measurable component) and appear only in the consequent of rules;
- The inner variables can appear on both sides of fuzzy rules, and they are used to represent mutual regulations among the system's components.

The state of the input variables is time-dependent, controlled by means of user-defined functions that influence the evolution over time of the whole system. On the contrary, the state of all other variables change according to the synchronous evaluation of the fuzzy rules.

In this work, we exploit a 0-order Sugeno inference engine to update the state of the variables, as implemented in the Simpful library (Spolaor et al., 2020a). To evaluate the next state of a variable over time, the Sugeno method performs an aggregation of the output values produced by the rules, weighted according to the firing degrees of the antecedents of each rule (see Sugeno, 1985 for additional information). After the Sugeno inference, the state of all non-input variables is updated and the dynamic simulation of the DFM proceeds to the following time step. The process is iterated until the simulation time reaches the user-set maximum value *t*_*max*_.

Given a DFM, an elementary perturbation of the system is defined as a tuple $\pi =\left(L,F,t_{b},t_{e}\right)$, where L is a linguistic variable whose state in π is set to the value calculated using the time-dependent function F along the perturbation interval (*t*_*b*_, *t*_*e*_), where 0 ≤ *t*_*b*_ < *t*_*e*_ ≤ *t*_*max*_. Multiple perturbations can be applied to the system in order to simulate the effect of multiple and simultaneous treatments: in this case, the global perturbation of the system is a defined as a list of *k* elementary perturbations Π = (π_0_, …, π_*k*_). It is worth noting that, during a perturbation, the Sugeno inference is disabled and the state update is performed only by the function F for any perturbed variable L. We refer the interested reader to (Nobile et al., 2020) for additional details about the definition, simulation and analysis of DFMs.

### 2.2. NSGA-II

DFMs can involve a large number of variables, leading to a combinatorial explosion of the possible system's perturbations to be tested. Global optimization algorithms can be employed to efficiently explore this huge space of perturbations, with the advantage of limiting the amount of simulations needed to test the system's response to any specific perturbation. In Nobile et al. (2020), for instance, the optimization task of the DFM of K-ras cancer cells was executed by means of Simulated Annealing (SA), a single-objective meta-heuristics proposed by Kirkpatrick et al. (1983). Specifically, the fitness function exploited in SA relied on a very simple penalty factor to limit the number of elementary perturbations appearing in the final solution. So doing, we could identify optimal solutions that correspond to simple drug combinations.

In this work, we consider a different approach for the prediction of combination therapies, using the Non-dominated Sorting Genetic Algorithm (NSGA-II), a population-based multi-objective elitist global optimization method proposed by Deb et al. (2002). The multi-objective approach is aimed at identifying the so-called Pareto front of dominant solutions, both from the point of view of the treatment *effectiveness* and of the *number of perturbations* (see section 4). A solution is said to be Pareto-dominant when there are Ω conflicting objectives to be optimized and no objective (e.g., effectiveness) can be improved without affecting at least another one (e.g., number of perturbations). As in the case of other multi-objective meta-heuristics, in NSGA-II the population P of candidate solutions converges to the Pareto front of dominant solutions, i.e., the set of optimal solutions that cannot be further improved without affecting one of the Ω objectives.

Formally, domination is defined as follows: a solution $y_{1}\in P$ dominates^1^ another solution $y_{2}\in P$ if:

- *f*_*i*_(*y*_1_) ≤ *f*_*i*_(*y*_2_) for all *i* ∈ {1, …, Ω};
- *f*_*j*_(*y*_1_) < *f*_*j*_(*y*_2_) for at least one index *j* ∈ {1, …, Ω},

where *f*_*k*_, for *k* = *i,j*, denotes the *k*-th objective (also named fitness function).

Thus, given an arbitrary population P, a ranking of non-dominated solutions can be calculated in order to obtain a list of Pareto fronts. Pareto dominance induces a partial ordering on the candidate solutions, and NSGA-II exploits this ordering to perform dominance-based selection and crossover.

The functioning of NSGA-II can be summarized as follows. The optimization process starts by generating a random population P consisting in *Q* individuals; then, at the beginning of each generation, NSGA-II calculates a merged population $P^{merge}=P\cup E$, corresponding to the union of the current population P and the set E of dominant individuals found so far. Non-dominated sorting is performed to obtain the Pareto fronts in $P^{merge}$. Individuals belonging to the best non-dominated sets are directly selected and added to a temporary population $P^{temp}$ for the application of genetic operators. Since it is generally the case that $\left|P^{merge}|>|P\right|$, some individuals of one of the Pareto fronts cannot be directly selected to be included in $P^{temp}$, to prevent an excessive amount of individuals in the next generation. In this case, NSGA-II limits the new population to exactly *Q* individuals by selecting only a subset of the individuals of this front. Such selection is carried out by using a crowded-comparison operator ≺_*n*_, which calculates the crowding distance of each putative solution. Thanks to the crowding ranking, NGSA-II can deterministically select the most “diverse” individuals and complete the generation of the new population, maintaining a high level of diversity in the population. As soon as $P^{temp}$ is composed by *Q* individuals, NSGA-II applies the crossover and mutation operators to create the next generation $P^{new}$ of candidate solutions that will replace the previous population P. The process iterates until a halting criterion is met; in this work, the algorithm stops after a fixed number of generations *IT*_max_. A schematic representation of the functioning of NSGA-II is shown in Supplementary Figure 1.

## 3. Multi-Objective Identification of Optimal Treatments

**Figure 1:**
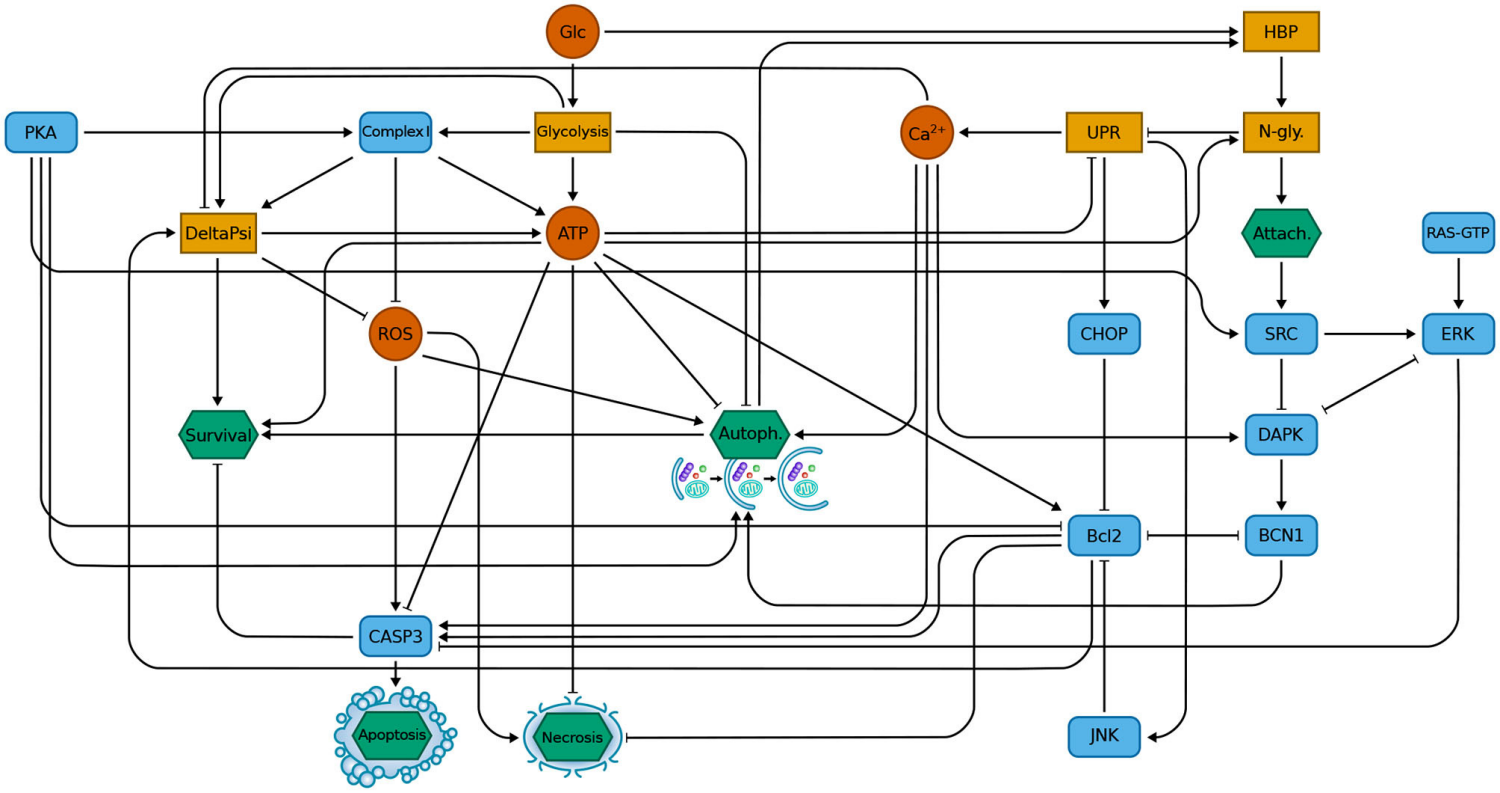
The interaction map of the programmed cell death model, showing the mutual regulation of the components. In the figure, single molecules are represented by red circles; blue rounded rectangles represent proteins; orange rectangles denote processes; green hexagons represent the outputs of the model. Positive and negative regulations are denoted as arrows and blunt-ended arrows, respectively. Figure adapted from Nobile et al. (2020).

In this work, we exploit the DFM of programmed cell death in K-ras cancer cells defined in Nobile et al. (2020) as a case in study to test the effectiveness of the multi-objective approach. This model describes the behavior of tumor cells in glucose starvation, and it is characterized by the presence of heterogeneous interacting components ranging from ions (e.g., calcium), metabolites (e.g., ATP), proteins (e.g., BCN1, Bcl2), cellular processes (e.g., unfolded protein response), and phenotypes (i.e., survival, apoptosis, necrosis, autophagy, and attachment). Moreover, the model describes the role of protein kinase A (PKA) in promoting cancer cell survival during glucose starvation. PKA corresponds to an input variable that can be set either to the “low” or to the “high” state, in order to mimic its inactivation or hyperactivation, respectively (see Nobile et al., 2020 for more information). The model was previously validated against data obtained from both mouse fibroblasts transformed by oncogenic K-ras expression (NIH3T3 K-ras cells) and a human K-ras-mutated breast cancer cell line (MDA-MB-231). A schematic representation of the components involved in the model and their interactions is shown in Figure 1.

The main goal of this work is to present a novel evolutionary methodology for the automatic identification of optimal treatments, able to induce programmed cell death in cancer cells, while keeping the complexity of the treatment (i.e., the number of perturbations) under control. In this context, a treatment is modeled as a set of simultaneous perturbations of the DFM. A perturbations is applied throughout the whole simulation, i.e., from *t*_*b*_ = 0 to *t*_*e*_ = *t*_*max*_. The identification of such treatments can be formulated as a multi-objective combinatorial optimization problem, which is here tackled using NSGA-II.

Each individual of the NSGA-II population represents a putative global perturbation Π of the system, encoded as a sequence of *D* symbols from the alphabet Σ = {0, 1, 2}, where 0 means “unperturbed,” while 1 and 2 encode the “low” and “high” linguistic terms, respectively. The “low” term represents the inhibition of a system's component, while “high” represents its overexpression/hyper-activation. In this work, the solutions have length *D* = 16, since the following variables of the model can be perturbed: Attachment, Autophagy, BCN1, Bcl2, CI, Ca^2+^, CHOP, DAPK, DeltaPsi, ERK, HBP, JNK, N-glycosylation, ROS, Src, and UPR.

NSGA-II explores the search space of possible perturbations, looking for the Pareto front of the most effective treatments. Specifically, the following 3 objectives are considered in this work:

- *f*_↑*apo*_: maximize the state increase of the variable Apoptosis;
- *f*_↓*nec*_: minimize the state increase of the variable Necrosis;
- *f*_↓*com*_: minimize the complexity of the solution, which is calculated as the number of elementary perturbations in Π (i.e., the elements of the candidate solutions that are different from 0).

The effect of the perturbation on a variable is calculated as the difference between the initial state of that variable and the state after some time Δ. The effect of the crossover operator exploited by NSGA-II is to randomly exchange the perturbations between two selected individuals; the mutation operator randomly changes one or more elements of an individual by modifying the current symbol 0, 1, or 2 with another symbol in Σ.

Similarly to Nobile et al. (2020), we assume that Δ = 13, that is, the time point when half of the available glucose has been consumed by the cell. The evaluation interval of the perturbations, as well as the function employed to model Glucose consumption, are shown in Supplementary Figure 2. Formally:

- $f_{apo}\left(\pi \right)=x_{apo}^{\pi }\left(\Delta \right)-x_{apo}^{\pi }\left(0\right)$
- $f_{nec}\left(\pi \right)=x_{nec}^{\pi }\left(\Delta \right)-x_{nec}^{\pi }\left(0\right)$

where $x_{apo}^{\pi }\left(t\right)$ and $x_{nec}^{\pi }\left(t\right)$ denote, respectively, the simulated state of the Apoptosis and Necrosis variables, using perturbation π at the time step *t*.

The algorithm NSGA-II used in this work was implemented using the Platypus library (Platypus library, 2020), version 1.0.2. The DFM of K-ras cancer cells was implemented using the Simpful library (Spolaor et al., 2020a), version 2.0.10. Matplotlib version 3.1.3 and numpy version 1.18.1 were also used. The source code of the model and of the multi-objective optimization method is available on GitHub, under GPL license, at the following URL: https://github.com/sspola/DynamicFuzzyModels.

## 4. Results

In order to investigate the effectiveness of our computational methodology, we performed two different tests. In the first one we considered two objectives, i.e., maximizing the apoptosis while minimizing the complexity of the treatment (section 4.1). In the second one we introduced a third objective, i.e., the minimization of necrosis, in order to identify perturbations favoring a controlled programmed cell death while mitigating the inflammatory response (section 4.2). These two tests were performed in both PKA low and high conditions, in order to evaluate the effects of drug combinations when PKA is inactive as well as hyperactivated.

The settings used in the 2-objectives optimization are: *Q* = 100 individuals, *IT*_*max*_ = 100 iterations, corresponding to a budget of 10,000 DFM simulations for the fitness evaluation. The settings employed for the 3-objectives optimization are the same as above, with the exception of an increased number of iterations, i.e., *IT*_*max*_ = 150, corresponding to 15,000 DFM simulations for the fitness evaluations. All tests were performed exploiting Platypus' default settings for integer-valued problems; uniform initialization of individuals; tournament selection with size equal to 2; half uniform crossover (Picek and Golub, 2010); and bit flip mutation (Davis, 1991).

### 4.1. Optimization With 2 Objectives: *f*_↑*apo*_ and *f*_↓*com*_

**Figure 2:**
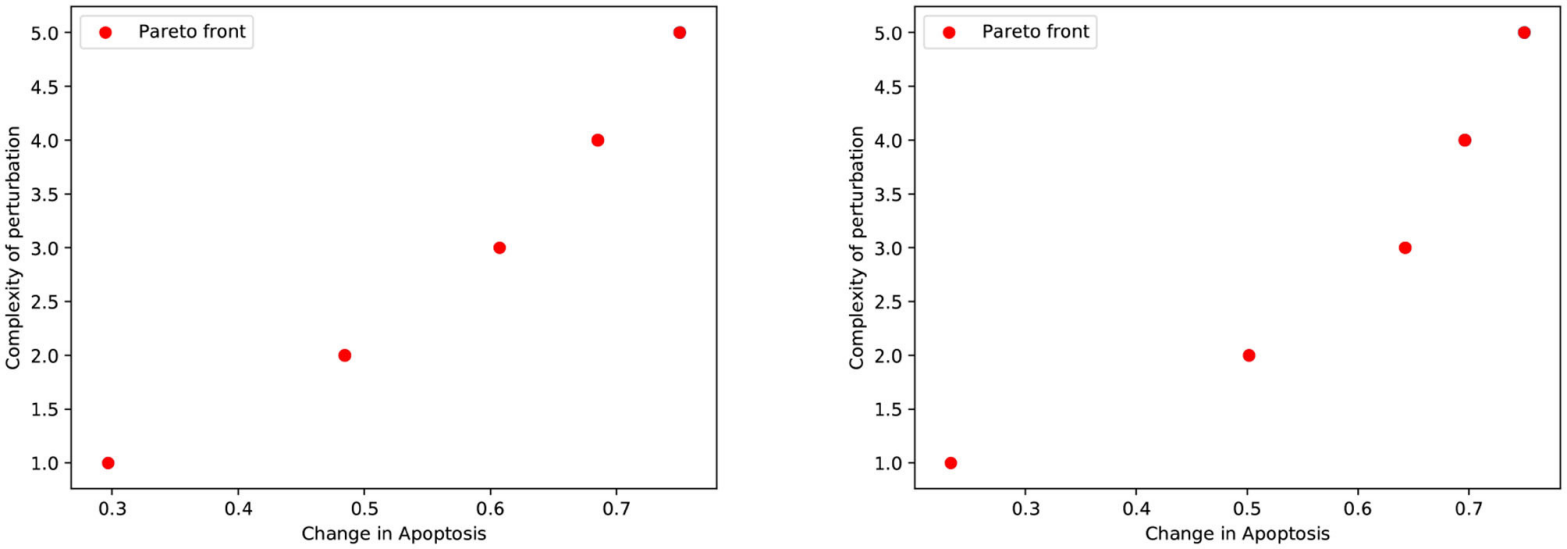
Pareto dominant solutions obtained by NSGA-II using 2 objectives (*f*_↑*apo*_ and *f*_↓*com*_) in PKA low **(left)** and high **(right)** conditions.

We first considered the multi-objective problem with 2 conflicting objectives, that is, maximizing the apoptosis (*f*_↑*apo*_) and minimizing the complexity of the treatment (*f*_↓*com*_). The Pareto fronts generated at the end of the NSGA-II optimization run in PKA low and high conditions are shown in Figure 2. Concerning the complexity of the solutions, it is worth noting that any set of perturbations including more than 3 elements would be difficult to implement both in the laboratory practice and as a therapy: specific drugs need to be used for each perturbed component, and their dose and administration protocols must be developed on cell cultures, a procedure that would lead to a combinatorial number of experimental validations. Moreover, an increase in the number of drugs might augment the chance of side effects at the cellular or the organism level. For these reasons, we decided to filter out from the Pareto front the optimal solutions consisting in more than 3 elements, and to maintain the six solutions reported in Table 1.

**Table 1:** Optimal perturbations found by NSGA-II, using 2 objective functions, in both PKA high and low conditions.

**Condition**	**Perturbation**
PKA low	Ca^2+^ is high Ca^2+^ is high, Bcl2 is low Ca^2+^ is high, Bcl2 is low, CI is low
PKA high	Bcl2 is Low ERK is low, UPR is high ERK is low, UPR is high, CI is low

#### 4.1.1. PKA Low

The solutions found for the PKA low condition consist of 3 components: Ca^2+^, Bcl2 and CI. Ca^2+^ represents the level of the calcium ions in the cytoplasm of the cell. In physiological conditions, calcium is stored in the mitochondria and in the endoplasmic reticulum. A release of calcium ions in the cytoplasm (“Ca^2+^ is high”) is connected to cellular stress, and leads to programmed cell death via apoptosis (Krebs et al., 2015). Bcl2 is a key protein regulator of programmed cell death, which binds and inhibits other pro-apoptotic proteins of the Bcl2 protein family; its inhibition (“Bcl2 is low”) allows the initiation of apoptosis (Borner, 2003). Complex I (CI) is an important protein complex responsible for energy production in the mitochondrion. Tumor cells that rely on glucose for their survival (i.e., cells displaying Warburg effect, Vander Heiden et al., 2009) preferentially produce energy via glycolysis instead of employing mitochondria and CI. However, the disruption of CI in these cells creates harmful oxidative species that induce stress and trigger apoptotic processes. Thus, its inhibition (“CI is low”) favors apoptosis (Palorini et al., 2013).

**Figure 3:**
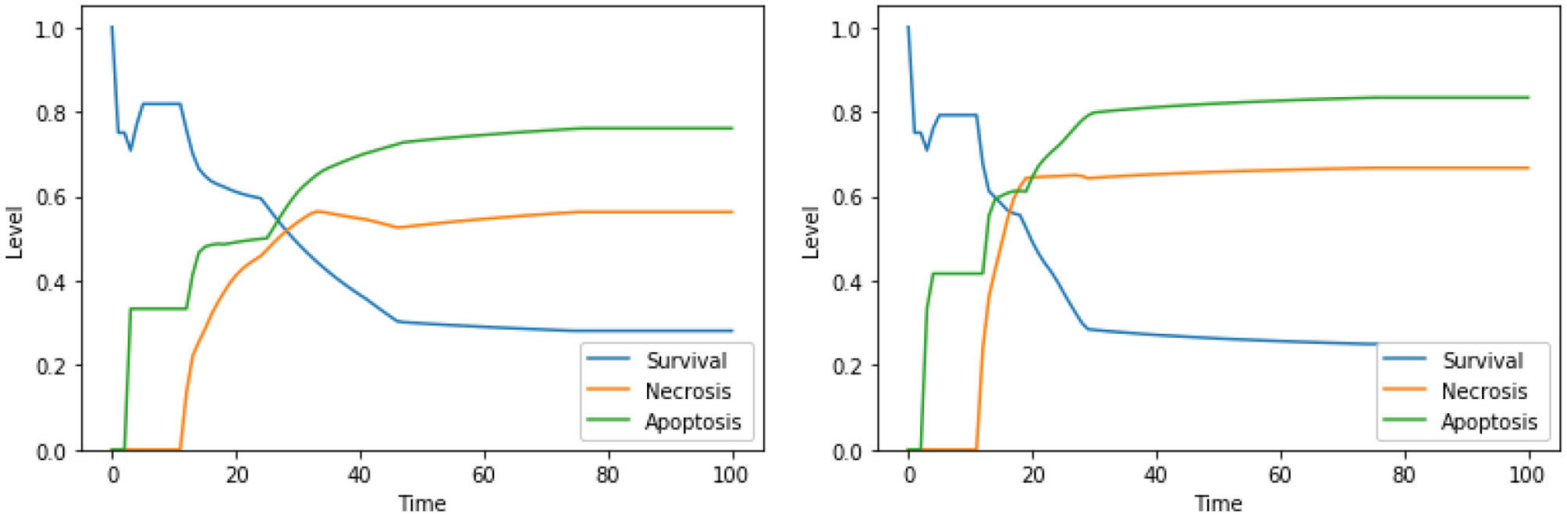
Simulation of the DFM in the PKA low condition, using the perturbations “Ca^2+^ is high, Bcl2 is low” **(left)** and “Ca^2+^ is high, Bcl2 is low, CI is low” **(right)**.

**Figure 4:**
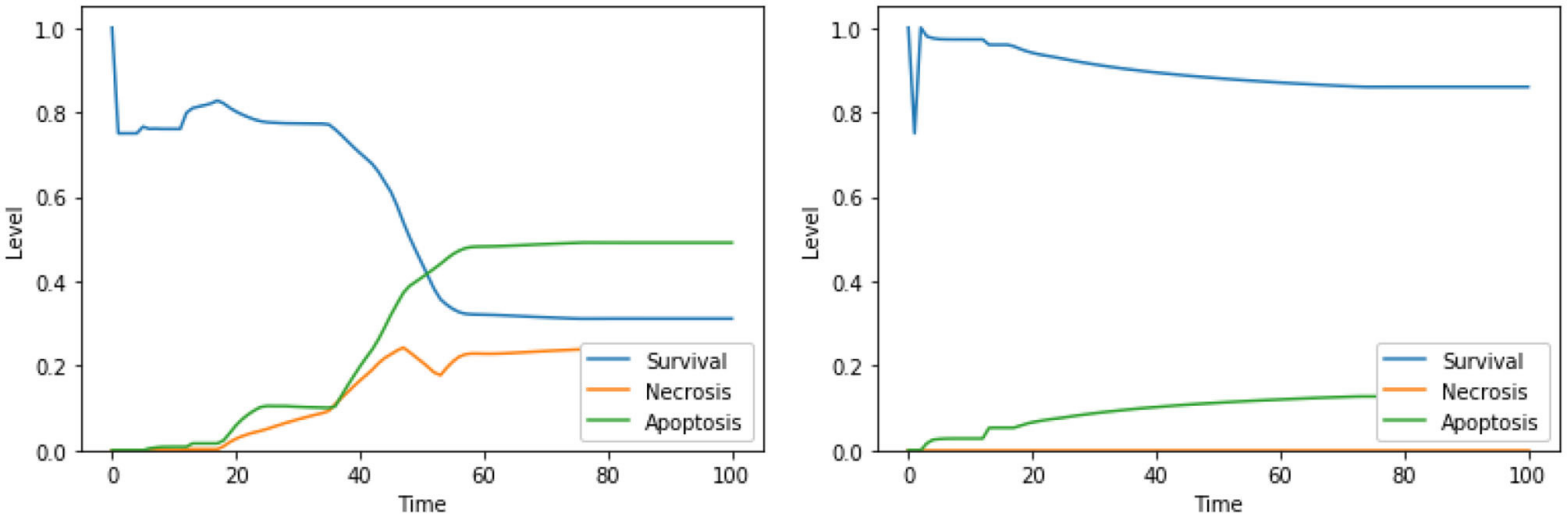
Dynamics of the unperturbed DFM, in the case of low **(left)** and high **(right)** PKA expression.

Perturbations involving one among Ca^2+^, Bcl2, or CI are known to trigger apoptosis, and were analyzed before (Borner, 2003; Li et al., 2003; Krebs et al., 2015) confirming the validity of our findings; however, only few studies investigated the outcome of the combinations of such perturbations. The interactions between cytoplasmic calcium level and Bcl2 were object of study in several cell types (Vervliet et al., 2016). On the contrary, to the best of our knowledge, the combined effect of high calcium level in the cytoplasm, inhibition of Bcl2 and inhibition of CI is, and will require an experimental validation that is beyond the scope of this work. According to the dynamics generated by our model, both the set of perturbations “Ca^2+^ is high, Bcl2 is low” (Figure 3, left panel) and “Ca^2+^ is high, Bcl2 is low, CI is low” (Figure 3, right panel) increase the level of apoptosis with respect to the unperturbed condition (see Figure 4, left panel, for a comparison). It is also worth noting that the perturbation involving 3 elements slightly increases the level of apoptosis with respect to the perturbation involving 2 elements, at the cost of a significant increase of necrosis.

#### 4.1.2. PKA High

In addition to the Bcl2 and CI components identified in the case of PKA low condition, the perturbations found for the PKA high condition include the ERK protein and the Unfolded Protein Response (UPR). ERK is a signaling protein involved in the regulation of cell division; its inhibition (“ERK is low”) facilitates the arrest of cell cycle and the initiation of apoptosis (Mendoza et al., 2011), which is fundamental as the signaling of cell division is altered in tumor cells. The UPR is a cellular process that is activated when the cell faces severe stress, caused for example by the lack of nutrients or the presence of oxidative species. The sustained activation of this process (“UPR is high”) induces apoptotic cell death (Hetz, 2012).

**Figure 5:**
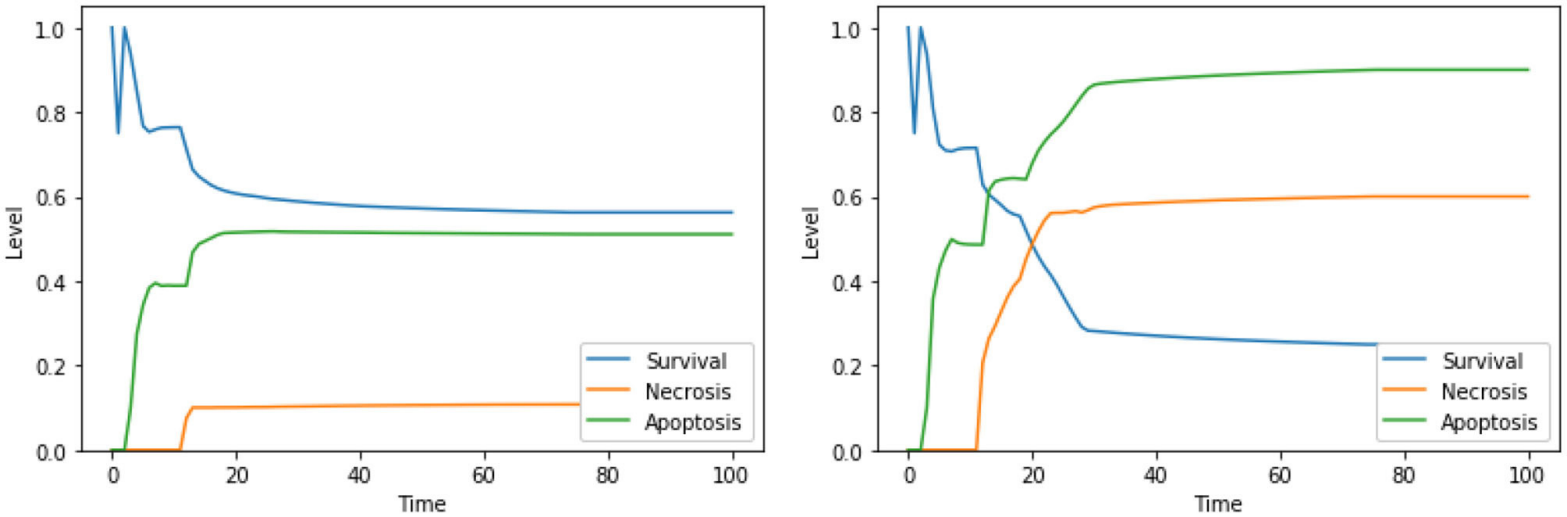
Simulation of the DFM in the PKA high condition, using the perturbations “ERK is low, UPR is high” **(left)** and “ERK is low, UPR is high, CI is Low” **(right)**.

As mentioned above, single perturbations of Bcl2, ERK, UPR, and CI have been studied before, given their role in regulating survival and programmed cell death. The disruption of mitochondrial membrane potential (possibly via CI impairment) combined with the hyperactivation of UPR was already found as a promising target combination in Nobile et al. (2020), and the effects of such perturbation were proved in Liao et al. (2017) and Szebeni et al. (2017). The interactions between the concurrent inhibition of ERK and activation of UPR were also investigated (Hu et al., 2004), but, to the best of our knowledge, no therapies based on this perturbations exist. Notably, according to the dynamics obtained with our DFM, this perturbation increases the level of apoptosis significantly (Figure 5, left panel) with respect to the unperturbed condition (see Figure 4, right panel, for comparison). The solution consisting of 3 perturbed components (“ERK is low, UPR is high, CI is low”) further increases the level of apoptosis (Figure 5, right panel), once again at the cost of a significant increase of the necrosis level. To the best of our knowledge, the effectiveness of this three targets combination in inducing cancer cell death has never been studied, and it deserves additional experimental validation to assess its ability in inducing apoptotic and/or necrotic cell death (that is beyond the scope of this work).

### 4.2. Optimization With 3 Objectives: *f*_↑*apo*_, *f*_↓*nec*_, and *f*_↓*com*_

When designing combination therapies, necrotic cell death should be avoided since necrosis causes the release of cellular contents in the extracellular space, triggering inflammation and leading to tissue damage (Kroemer et al., 2009). Moreover, despite the results obtained in the case of the 2 objectives *f*_↑*apo*_ and *f*_↓*com*_ suggest novel putative targets for combination therapies, the necrosis increase obtained as a counter effect cannot be neglected. These considerations led us to perform an additional analysis, adding the third objective *f*_↓*nec*_.

**Figure 6:**
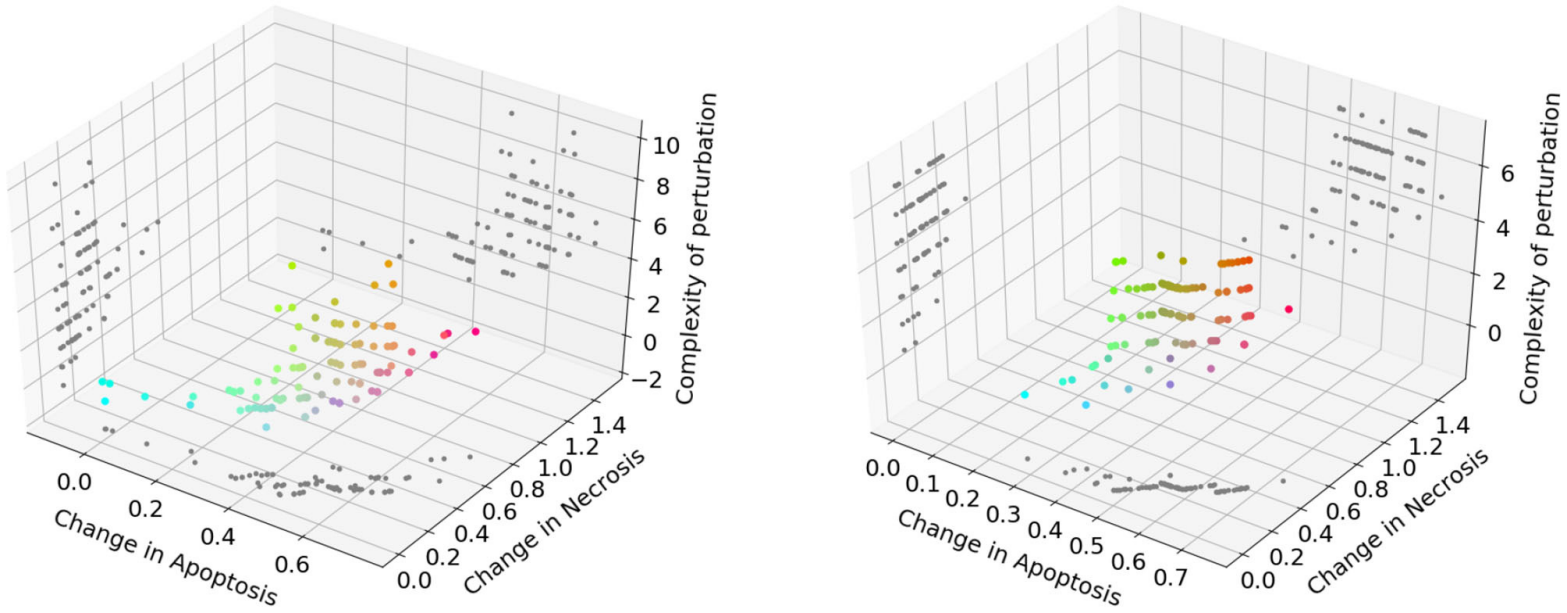
Pareto dominant solutions obtained by NSGA-II using 3 objectives (*f*_↑*apo*_, *f*_↓*nec*_, and *f*_↓*com*_) in the PKA low condition **(left)** and PKA high condition **(right)**. Solutions' color is represented by RGB triplets, according to their fitness values (i.e., red as change in apoptosis, green as change in necrosis and blue as complexity of the solution). Projections of the solutions on 2D planes are represented in gray.

The Pareto fronts generated at the end of the NSGA-II optimization run in PKA low and high conditions are shown in Figure 6 (left and right panel, respectively). Following the reasoning adopted in the previous analysis, we excluded solutions consisting in more than 3 perturbed elements, obtaining 31 unique solutions for the PKA low condition, and 13 for the PKA high condition. The complete list of solutions for both conditions is given in Supplementary Tables 1, 2.

Given the large number of optimal solutions found, we performed some additional filtering steps to exclude non-novel and non-interesting solutions and perturbations that might result in harmful treatments.. We inspected each solution by analyzing both its fitness value for the 3 objectives and the dynamics obtained by simulating that perturbation with the DFM, in order to identify a subset of solutions that:

- Do not include only a single perturbed element, since single perturbations of each element of our models have been already extensively studied in the literature;
- Do not involve elements belonging to the same pathway and/or immediately adjacent in the model (see Figure 1);
- Do not include perturbations that are difficult to implement or harmful for the organism, that is, solutions containing an increase in ROS (“ROS high”) or an hyperactivation of the Src oncogene (“Src high”);
- Are characterized by a low value of *f*_↓*nec*_. These solutions are generated in the extreme part of the Pareto front (i.e., they have non-optimal values of *f*_↓*nec*_, but they reach optimal values of *f*_↑*apo*_ and *f*_↓*com*_), but they are not interesting for the purpose of this 3 objectives analysis, that is, identifying solutions with a low increase in necrosis.

The remaining subset of solutions that were deemed to be the most promising is listed in Table 2.

**Table 2:** Optimal perturbations found by NSGA-II, using 3 objective functions, in both PKA high and low conditions.

**Condition**	**Perturbation**
PKA low	Src IS low, UPR IS high CI IS low, UPR IS high DeltaPsi IS low, UPR IS high ERK IS low, UPR IS high Ca^2+^ IS high, ERK IS low, UPR IS high DeltaPsi IS low, ERK IS low, UPR IS high CI IS high, ERK IS low, UPR IS high CI IS low, ERK IS low, UPR IS high
PKA high	ERK IS low, UPR IS high DAPK IS low, UPR IS high DeltaPsi IS low, UPR IS high Ca^2+^ IS high, ERK IS low Ca^2+^ IS high, ERK IS low, N-glycosylation IS low DeltaPsi IS low, ERK IS low, UPR IS high

#### 4.2.1. PKA Low

In addition to the model components pertaining the solutions found in the previous 2-objectives analysis, in the case of the PKA low condition, Src and DeltaPsi are here suggested as possible therapeutic targets. Src is a tyrosine kinase, involved in the control of several signaling pathways regulating cell proliferation, differentiation, and motility. It plays a role in the development of several cancer types, and its inhibition (“Src is low”) is known to be effective in stopping cancer cell proliferation (Yeatman, 2004). DeltaPsi represents the mitochondrial membrane potential, that is, the transmembrane potential existing across the mitochondrial membrane. The maintenance of this potential is crucial for cell survival, and its loss (“DeltaPsi is low”) initiates apoptotic processes (Zorova et al., 2018).

The first solution, “Src IS low, UPR IS high,” confirms the validity of our computational approach. As a matter of fact, a vast body of literature and studies exist regarding therapies based on Src inhibition, and its connections with the UPR—as encoded in the solution—have also been studied before (Fan et al., 2013; Yu et al., 2020).

By inspecting the solutions listed in Table 2, we can deduce that our model suggests a crucial role of UPR in promoting a high level of apoptosis, while keeping necrosis at a low level. Indeed, UPR appears in all solutions found for the PKA low condition, and also in most of the solutions found for PKA high.

**Figure 7:**
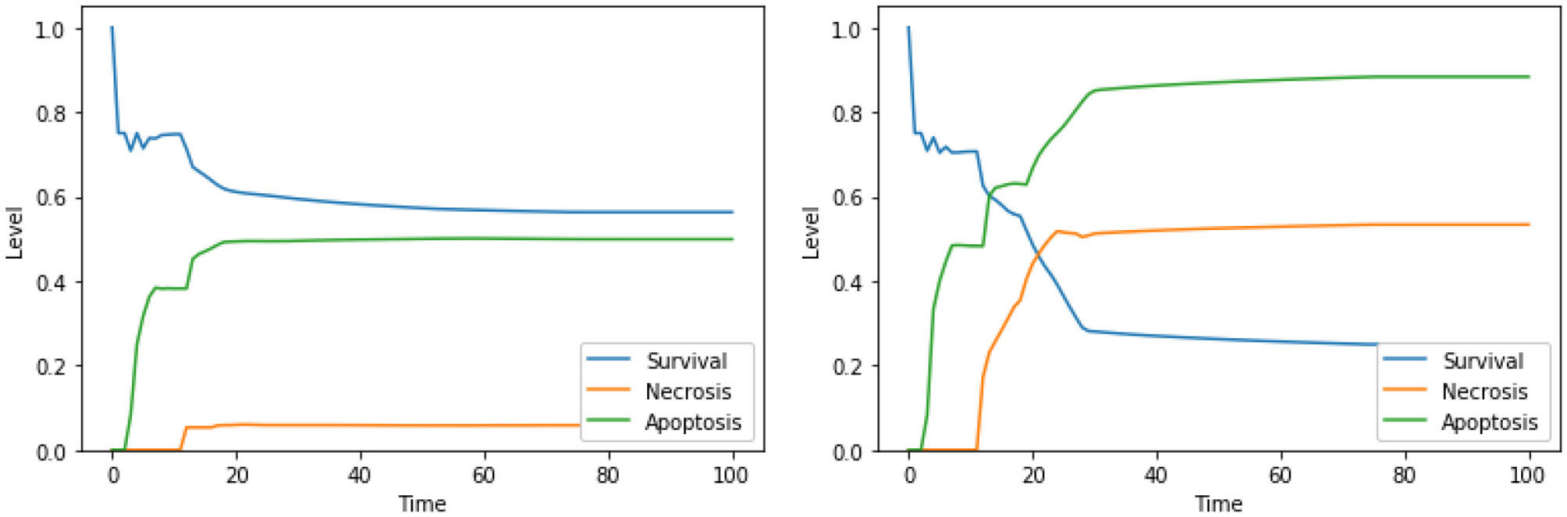
Simulation of the DFM in the PKA low condition, using the perturbations “CI IS high, ERK IS low, UPR IS high” **(left)** and “CI IS low, ERK IS low, UPR IS high” **(right)**.

Moreover, our model suggests a protective role of a functional CI toward both apoptosis and necrosis, a phenomenon that was already experimentally observed in Nobile et al. (2020) and Palorini et al. (2013). This behavior can be observed when comparing the dynamics obtained with the solutions “CI IS high, ERK IS low, UPR IS high” and “CI IS low, ERK IS low, UPR IS high,” shown in Figure 7. The inhibition of CI (Figure 7, right panel) leads to a higher level of apoptosis, at the cost of a significant increase in the level of necrosis, a behavior that is reproduced with minimal differences also in the case of the solution “CI IS low, UPR IS high” (dynamics not shown). Given the role of CI in the avoidance of necrosis, we argue that disruption of the mitochondrial membrane potential (DeltaPsi), while keeping the functional state of CI intact, might be a better target for therapy, as highlighted in what follows by the other solutions containing this system's component.

**Figure 8:**
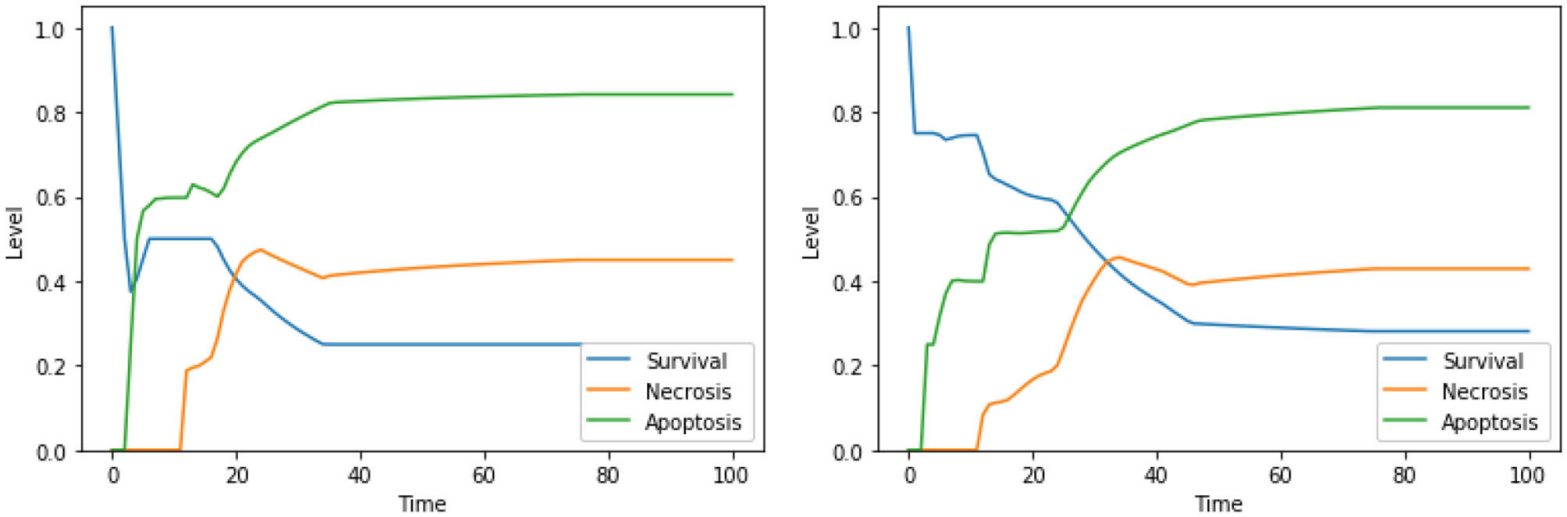
Simulation of the DFM in the PKA low condition, using the perturbations “DeltaPsi IS low, ERK IS low, UPR IS high” **(left)** and “Ca^2+^ IS high, ERK IS low, UPR IS high” **(right)**.

The solution “DeltaPsi IS low, UPR IS high” was already predicted in the analysis performed in Nobile et al. (2020), and it has been experimentally validated in previous studies (Liao et al., 2017; Szebeni et al., 2017). The addition of ERK inhibition, as in the solution “DeltaPsi IS low, ERK IS low, UPR IS high,” leads to a significant increase in the level apoptosis when compared to the unperturbed condition, especially in the condition of a high availability of glucose (i.e., at the beginning of the simulation), as it can be seen in the left panel of Figure 8. The solution “ERK IS low, UPR IS high” was already obtained in the analysis exploiting 2 objectives, as discussed in section 4.1, albeit in the PKA high condition. We argue that the solutions “ERK IS low, UPR IS high” and “Ca^2+^ IS high, ERK IS low, UPR IS high” might as well represent good alternative therapies, displaying a significant increase in the level of apoptosis (the dynamics obtained with the latter solution are reported in Figure 8, right panel). In particular, it should be noted that the solutions listed above that are characterized by 3 perturbed elements target different cellular processes and organelles (e.g., mitochondria, endoplasmic reticulum, signaling pathways connected to the extracellular environment). According to our model, these perturbations are the most effective in increasing the apoptosis level. However, to the best of our knowledge, there are no studies available in the literature that tested these target combinations on cell cultures, possibly due to the hindrance to performing laboratory experiments comprising three different drugs.

#### 4.2.2. PKA High

For the PKA high condition, the optimal solutions also include the components DAPK and N-glycosylation. DAPK is a kinase involved in the regulation of several types of programmed cell death. It shows a dual role as an inducer of both apoptotic and survival processes (de Diego et al., 2010; Zhao et al., 2015). In our model, its inhibition (“DAPK is low”) results in the suppression of necrosis. N-glycosylation consists in the attachment of oligosaccharide chains to proteins in the endoplasmic reticulum. This process is pivotal for a correct protein folding of extracellular proteins and mediates cellular attachment, thus its disruption (“N-glycosylation is low”) leads to a decrease in cell survival (Gu et al., 2012).

**Figure 9:**
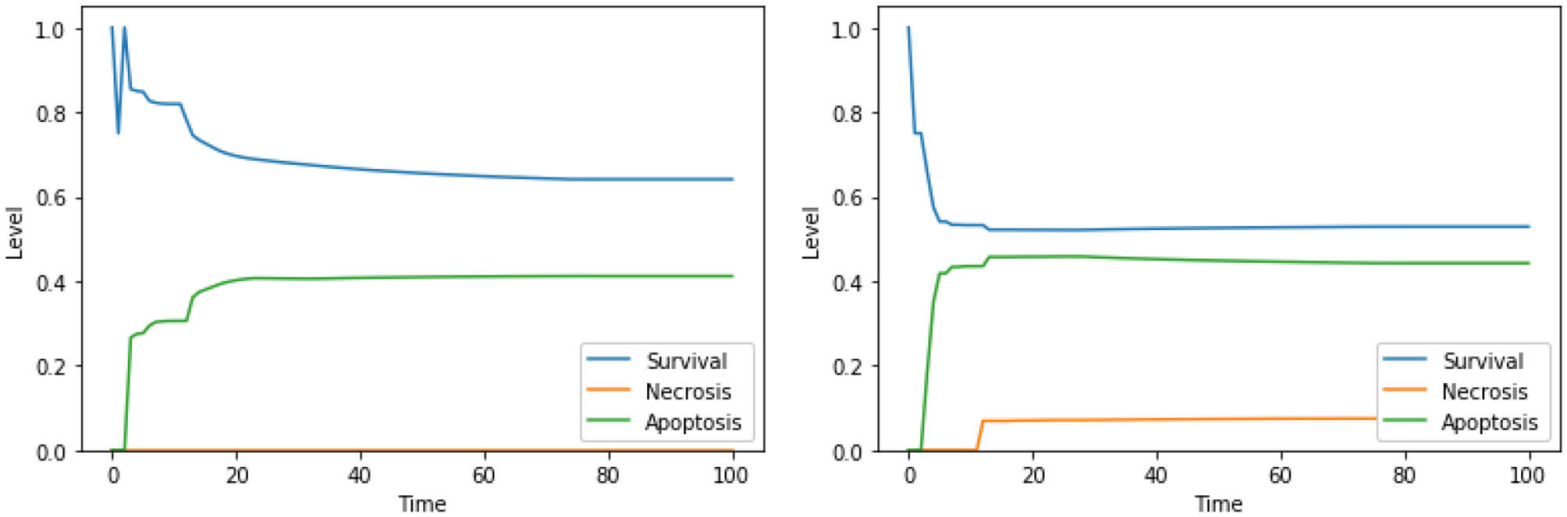
Simulation of the DFM in the PKA high condition, using the perturbations “Ca^2+^ IS high, ERK IS low” **(left)** and “DeltaPsi IS low, UPR IS high” **(right)**.

The solution “ERK IS low, UPR IS high” was obtained also in the analysis exploiting 2 objectives, as discussed in section 4.1. The solutions “DAPK IS low, UPR IS high,” “DeltaPsi IS low, UPR IS high,” and “Ca^2+^ IS high, ERK IS low” show a significant increase in apoptosis with respect to the unperturbed condition (Figure 4, right panel). We did not find any experimental work involving a simultaneous inhibition of DAPK and hyperactivation of UPR (as suggested by “DAPK IS low, UPR IS high”), thus this solution might represent a novel target combination. Disruption of mitochondrial membrane potential and hyperactivation of UPR (“DeltaPsi IS low, UPR IS high”) has already been validated as a promising anti-cancer therapy, as discussed in sections 4.1.2, 4.2.1 and in Liao et al. (2017), Szebeni et al. (2017), Nobile et al. (2020). The effects of the interplay between cytosolic levels of Ca^2+^ and the activation of ERK (“Ca^2+^ IS high, ERK IS low”) have been partially characterized in different cell lines (Rodriguez-Mora et al., 2005), but, to the best of our knowledge, they did not result in any therapy approach. Notably, “Ca^2+^ IS high, ERK IS low” (Figure 9, left panel) maintains the level of necrosis to 0, while the dynamics obtained with “DeltaPsi IS low, UPR IS high” (Figure 9, right panel) reach a higher value of both apoptosis and necrosis, with respect to the other two solutions.

**Figure 10:**
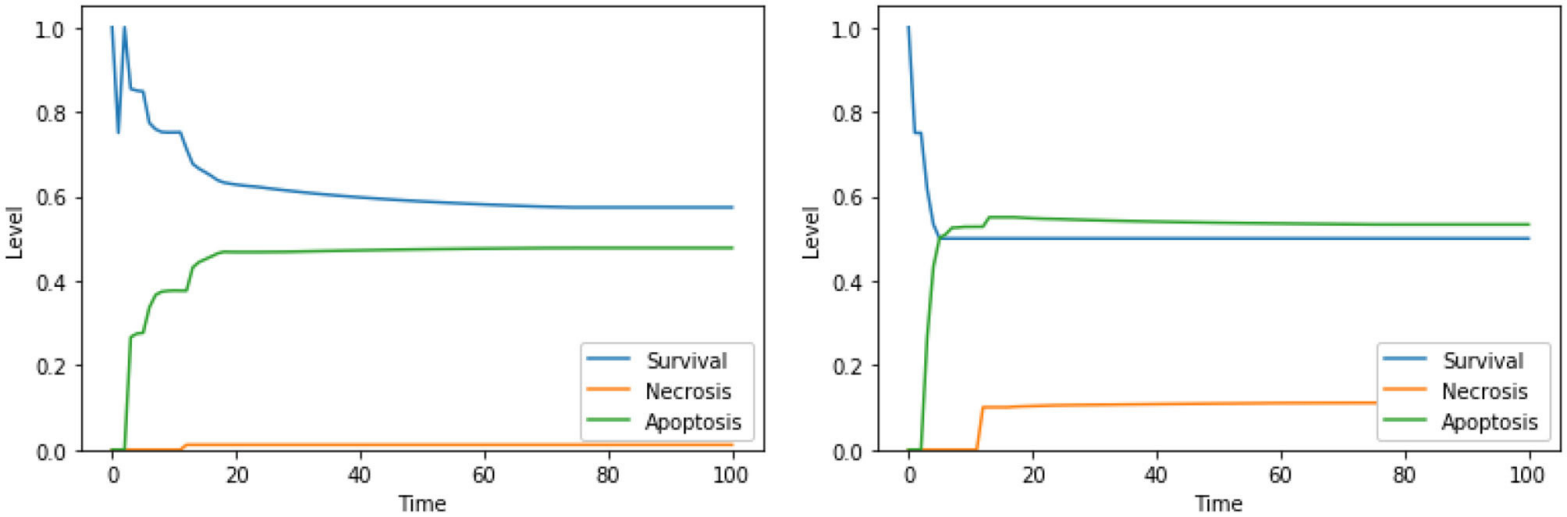
Simulation of the DFM in the PKA high condition, using the perturbations “Ca^2+^ IS high, ERK IS low, N-glycosylation IS low” **(left)** and “DeltaPsi IS low, ERK IS low, UPR IS high” **(right)**.

Similarly to what was already observed in the case of the PKA low condition, the solutions consisting of 3 elements lead to higher values of apoptosis with respect to solutions consisting of 2 elements, especially in the condition of a high availability of glucose (i.e., at the beginning of the simulation). This can be observed for “Ca^2+^ IS high, ERK IS low, N-glycosylation IS low” in Figure 10, left panel, and for “DeltaPsi IS low, ERK IS low, UPR IS high” in Figure 10, right panel. As previously stated, given that testing combinations of three drug targets in cell cultures poses considerable challenges, no experimental validation is available in the literature for these solutions. However, based on our results, we argue that the perturbations characterized by 3 elements, targeting different cellular processes and organelles, are the most effective in increasing apoptosis level in the PKA high condition as well, and they are worth additional experimental testing in the future.

## 5. Conclusions

In this work, we presented a novel computational approach to discover new putative combination therapies for cancer treatment, by exploiting dynamic fuzzy modeling and the NSGA-II algorithm for multi-objective optimization. Our results show that our approach can generate model perturbations (i.e., combination of drug targets) that were previously described in the literature as effective in inducing apoptosis, as well as novel ones. Their effects on the dynamic behavior of the DFM of oncogenic K-ras cancer cells suggest that such perturbations are effective in inducing apoptosis in this type of cancer cells. Worth of notice, higher values of apoptosis were obtained when the perturbation involves the impairment of different cellular processes, while lower values of necrosis were obtained by introducing a third objective in the optimization phase. We envision that this novel computational approach could be used to efficiently identify, develop and test new combination therapies for different types of cancer or other complex diseases (e.g., neurodegeneration) by exploiting existing FDA-approved drugs.

As a future extension of this work, we plan to test the validity of the most promising solutions found with our method, by means of tailored experimental studies. Indeed, the main advantage of coupling computational and experimental analyses is that we can reduce the time and cost constraints of lab procedures. From the computational point of view, we will extend our method by implementing a strategy for the automatic selection of interesting solutions inside the Pareto front (such as, for example, the ones presented in Branke et al., 2004; Zio and Bazzo, 2011), in order to speed up the analysis and reduce human bias when dealing with large Pareto fronts. We also plan to define a new representation of the candidate solutions in NSGA-II, in order to generate only solutions with a user-defined number of perturbed elements. Additionally, we will test the performance that can be achieved by coupling DFMs with alternative multi-objective optimization strategy to identify new combination therapies, such as NSGA-III (Deb and Jain, 2013) and SPEA2 (Zitzler et al., 2001).

The multi-objective approach could also be applied to hybrid models, obtained by coupling DFMs and mechanistic models (Spolaor et al., 2020b), in order to integrate precise quantitative information about cellular components and processes when it is available, and to obtain more comprehensive cancer cell models (Spolaor et al., 2019).

## Data Availability Statement

The code and the model supporting the conclusions of this article are available at the following GitHub repository: https://github.com/sspola/DynamicFuzzyModels.

## Author Contributions

SS and MN conceived the method, planned the experiments, and supervised the research activity. SS, MS, and MN implemented the method. MS performed the experiments. SS analyzed the results. SS and MS prepared and created the figures. SS and MN wrote the first draft of the manuscript. MS, MF, PC, and DB critically reviewed and edited the manuscript. DB and MN acquired financial support for this work. All authors read and approved its final version.

## Conflict of Interest

The authors declare that the research was conducted in the absence of any commercial or financial relationships that could be construed as a potential conflict of interest.

## Footnotes

^1^We hereby assume that all objectives must be minimized. This formalization of domination can be straightforwardly modified to consider the case of the maximization of (some) objectives by changing the signs of their objective values.
