# Supplementary material for: Screening for Combination Cancer Therapies With Dynamic Fuzzy Modeling and Multi-Objective Optimization
Source: Front Genet. 2021 Mar 31;12:617935. doi: 10.3389/fgene.2021.617935 (PMC8044361; doi:10.3389/fgene.2021.617935)
Supplement: Supplementary file 1 [file Data_Sheet_1.PDF]

Supplementary Material

Screening for combination cancer therapies with dynamic fuzzy modeling and multi-objective optimization

Simone Spolaor, Martijn Scheve, Murat Firat, Paolo Cazzaniga, Daniela Besozzi, Marco S. Nobile

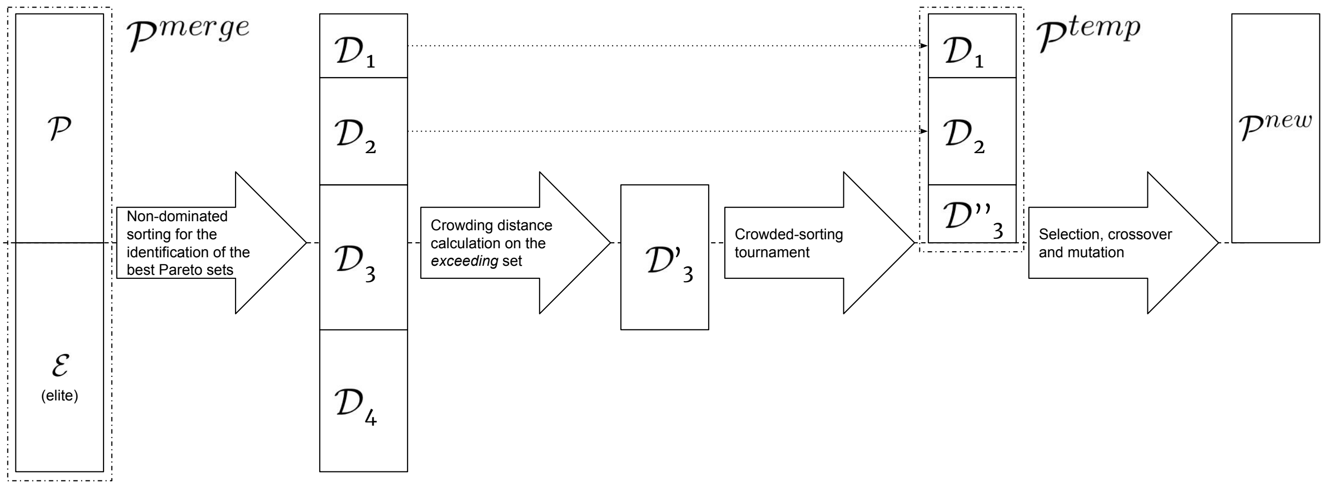

Figure S1. Schematic representation of the functioning of NSGA-II algorithm.

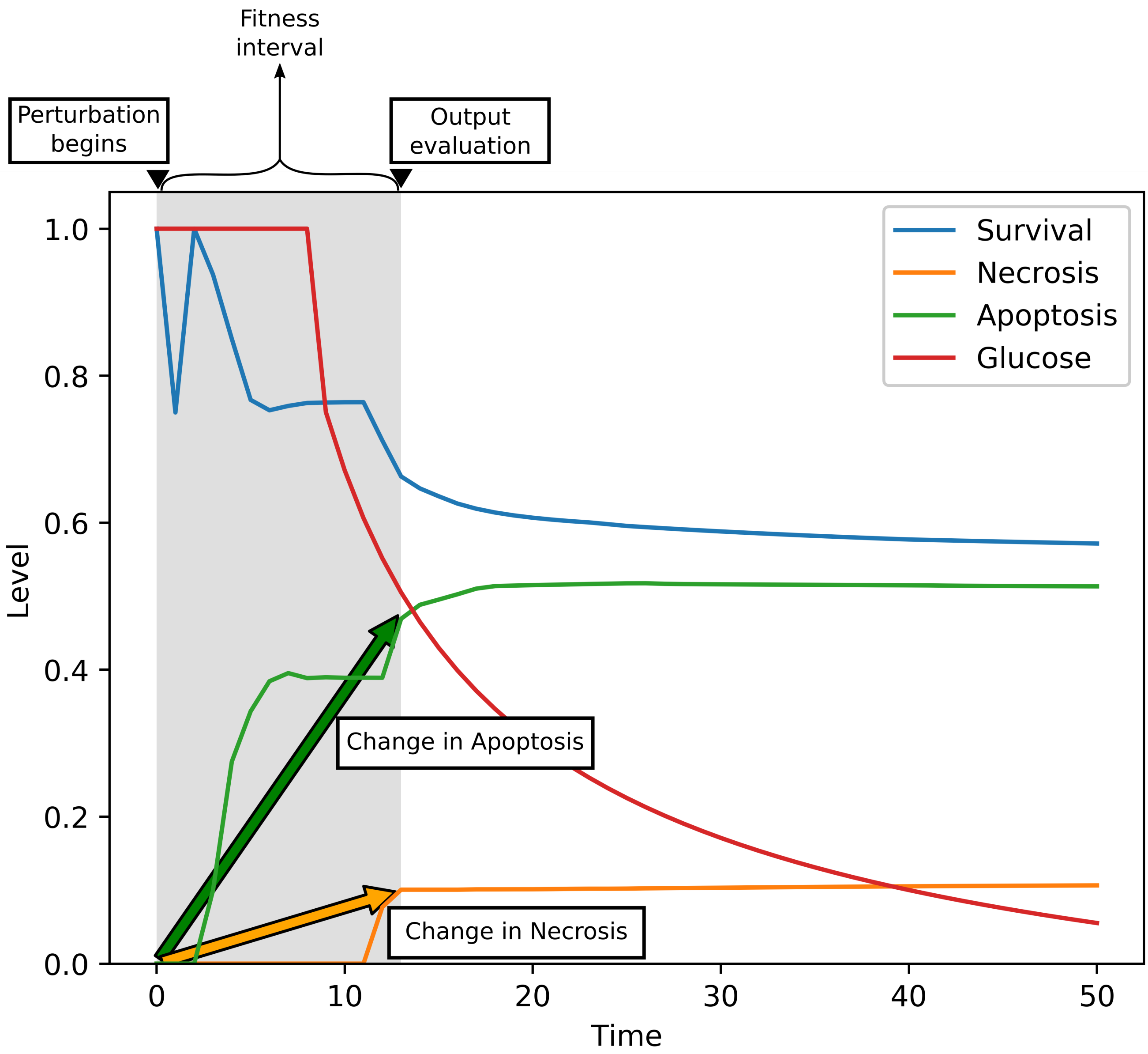

Figure S2. Evaluation of the changes in Apoptosis and Necrosis during the multi-objective optimization. The evaluation interval of the perturbation is highlighted by the grey area (in this case $\Delta = 13$).

SOLUTIONS OBTAINED IN THE 3 OBJECTIVES ANALYSIS

Table S1. Unique solutions for the 3-objective analysis with low PKA

Solution	Change in Apoptosis	Change in Necrosis	Complexity
CI IS high	0,0139	0,0000	1
ERK IS low	0,1275	0,0070	1
N-glycosylation IS low	0,2297	0,0584	1
CI IS high, Ca^{2+} IS high	0,2644	0,0000	2
Src IS low	0,0319	0,0047	1
ERK IS low, Src IS low, UPR IS high	0,5187	0,1270	3
CI IS high, DAPK IS low, UPR IS high	0,3630	0,0000	3
Src IS low, UPR IS high	0,4326	0,1040	2
UPR IS high	0,4243	0,0894	1
ERK IS low, UPR IS high	0,5145	0,1190	2
Ca^{2+} IS high, ERK IS low, UPR IS high	0,5145	0,1190	3
CI IS low, UPR IS high	0,5407	0,2835	2
Src IS high, UPR IS high	0,4160	0,0745	2
DeltaPsi IS low, ERK IS low, UPR IS high	0,6090	0,2202	3
ERK IS low, ROS IS high, UPR IS high	0,6167	0,3064	3
Ca^{2+} IS high, ERK IS low	0,3939	0,0468	2
ERK IS low, ROS IS low, UPR IS high	0,4656	0,0535	3
DAPK IS low, UPR IS high	0,4021	0,0697	2
CI IS high, DeltaPsi IS low, UPR IS high	0,4276	0,0187	3
CI IS high, ROS IS high, UPR IS high	0,5369	0,1754	3
ROS IS high, UPR IS high	0,5256	0,2815	2
CI IS high, Src IS high, UPR IS high	0,3746	0,0004	3
CI IS high, UPR IS high	0,3842	0,0187	2
N-glycosylation IS low, UPR IS high	0,4326	0,1040	2
DAPK IS low, ERK IS low, UPR IS high	0,4777	0,0697	3
Ca^{2+} IS high, UPR IS high	0,4243	0,0894	2
DeltaPsi IS low, UPR IS high	0,5175	0,1833	2
ERK IS low, Src IS high, UPR IS high	0,5069	0,1040	3
CI IS high, Ca^{2+} IS high, UPR IS high	0,3842	0,0187	3
CI IS high, ERK IS low, UPR IS high	0,4773	0,0535	3
CI IS low, ERK IS low, UPR IS high	0,6287	0,3106	3

Table S2. Unique solutions for the 3-objective analysis with high PKA

Solution	Change in Apoptosis	Change in Necrosis	Complexity
Ca^{2+} IS high, DeltaPsi IS low, ERK IS low	0,4568	0,0000	3
UPR IS high	0,4128	0,0693	1
Ca^{2+} IS high	0,2936	0,0000	1
Ca^{2+} IS high, ERK IS low	0,3851	0,0000	2
DeltaPsi IS low, ERK IS low, UPR IS high	0,5503	0,1006	3
ROS IS high, UPR IS high	0,5582	0,2174	2
DeltaPsi IS low, N-glycosylation IS low, UPR IS high	0,4633	0,0800	3
DAPK IS low, UPR IS high	0,3932	0,0357	2
Ca^{2+} IS high, ROS IS high, UPR IS high	0,5582	0,2174	3
ERK IS low, ROS IS high, UPR IS high	0,6426	0,2437	3
Ca^{2+} IS high, ERK IS low, N-glycosylation IS low	0,4574	0,0118	3
ERK IS low, UPR IS high	0,5018	0,1006	2
DeltaPsi IS low, UPR IS high	0,4571	0,0693	2
